# LB1579. A single and a two-dose regimen of MVA-BN elicit similar memory responses to a boost vaccination two years after the initial regimen, indicating potential flexibility for a delayed second dose of MVA-BN in the ongoing monkeypox emergency

**DOI:** 10.1093/ofid/ofac492.1889

**Published:** 2022-12-15

**Authors:** Heinz Weidenthaler, Florian Lienert, Daniela Reichhardt, Darja Schmidt, Günter Silbernagl, Thomas Meyer, Paul Chaplin

**Affiliations:** Bavarian Nordic, Fraunhoferstraße 13, Bayern, Germany; Bavarian Nordic, Fraunhoferstraße 13, Bayern, Germany; Bavarian Nordic, Fraunhoferstraße 13, Bayern, Germany; Bavarian Nordic, Fraunhoferstraße 13, Bayern, Germany; Bavarian Nordic, Fraunhoferstraße 13, Bayern, Germany; LMU University Hospital, Munich, Bayern, Germany; Bavarian Nordic, Fraunhoferstraße 13, Bayern, Germany

## Abstract

**Background:**

The ongoing monkeypox outbreak, a WHO-declared PHEIC, requires fast and broad rollout of MVA-BN vaccinations among risk groups. An initial single-dose strategy, followed by a delayed second dose beyond the approved 4-week interval, could help managing tight vaccine resources.

**Methods:**

Bavarian Nordic has run a study in vaccinia-naïve participants, randomized to receive either 1 MVA-BN vaccination (N = 181) or 2 MVA-BN vaccinations 4 weeks apart (N = 183). Of note, this study also evaluated participants with prior smallpox vaccination, who received 1 MVA-BN boost (N = 200). Subsets of approximately 75 participants of the vaccinia-naïve subjects having received either 1 or 2 doses of the vaccine were administered an MVA-BN boost 2 years later. Neutralizing antibodies (nAb) were measured using Vaccinia PRNT.

**Results:**

The 1xMVA-BN and 2xMVA-BN groups responded as expected to the primary regimen, with modest increases in nAb GMTs at Week 2 (5.1 and 4.8, respectively) that further increased at Week 4 (7.2 and 7.5). Two weeks after the second primary vaccination (at Week 6) (2xMVA-BN) nAb GMTs peaked (45.6) before stabilizing at Week 8 (34.0). In both the 1x and 2xMVA-BN groups, the nAb levels were near baseline 2 years after primary vaccination. After the MVA-BN boost at 2 years, both groups exhibited rapid anamnestic responses with nAb GMTs that peaked 2 weeks post-boost (80.7 and 125.3), at higher levels than those observed at any timepoint following primary vaccination, remaining elevated at 6 months post-boost (25.6 and 49.3). These anamnestic responses support the presence of durable immunological memory in both groups. No safety concerns were identified and the most common adverse event following a 2-year MVA-BN boost was injection site erythema in 82.2% of participants.

**Conclusion:**

A single dose of MVA-BN elicits a durable memory immune response for at least 2 years. Similarly, high nAb responses following a boost dose among subjects in the initial 1xMVA-BN or 2xMVA-BN groups indicate that the approved 4-week interval of the two-dose regimen could optionally be extended up to at least 2 years.

NCT00316524 and NCT00686582

**Disclosures:**

**Heinz Weidenthaler, MD**, Bavarian Nordic GmbH: Employee and received warrants **Florian Lienert, PhD**, Bavarian Nordic GmbH: employee Employee and received warrants **Daniela Reichhardt, PhD**, Bavarian Nordic A/S: employee Employee and received warrants **Darja Schmidt, Dr**, Bavarian Nordic GmbH: Employee and received warrants **Günter Silbernagl, MSc**, Bavarian Nordic: Employed|Bavarian Nordic GmbH: employee Employee and received warrants **Paul Chaplin, PhD**, Bavarian Nordic A/S: employee (CEO)|Bavarian Nordic A/S: Stocks/Bonds.

